# Supplementary material for: The intralumenal fragment pathway mediates ESCRT-independent surface transporter down-regulation
Source: Nat Commun. 2018 Dec 18;9:5358. doi: 10.1038/s41467-018-07734-5 (PMC6299085; doi:10.1038/s41467-018-07734-5)
Supplement: Supplementary file 2 — Description of Additional Supplementary Files [file 41467_2018_7734_MOESM2_ESM.pdf]

Description of Additional Supplementary Files

File Name: Supplementary Movie 1.
Description: *2-deoxyglucose triggers Hxt3-GFP internalization by vacuole fusion.*
Movie of a vacuole fusion event in a wild type cell demonstrating the formation of intralumenal fragment decorated with Hxt3-GFP after treatment with 2-deoxyglucose. Hxt3-GFP and FM4-64 fluorescence, and the two channels merged are shown. Scale bar and time are shown in Figure 2F.

File Name: Supplementary Movie 2.
Description: *Heat stress triggers Hxt3-GFP internalization by vacuole fusion.*
Movie of a vacuole fusion event in a wild type cell demonstrating the formation of intralumenal fragment decorated with Hxt3-GFP after heat stress. Hxt3-GFP and FM4-64 fluorescence, and the two channels merged are shown. Scale bar and time are shown in Figure 3E.

File Name: Supplementary Movie 3.
Description: *Cycloheximide induces Hxt3-GFP internalization by vacuole fusion.*
Movie of a vacuole fusion event in a wild type cell demonstrating the formation of intralumenal fragment decorated with Hxt3-GFP after cycloheximide treatment. Hxt3-GFP and FM4-64 fluorescence, and the two channels merged are shown. Scale bar and time are shown in Figure 4E.

File Name: Supplementary Movie 4.
Description: *Hxt3-GFP internalization after heat stress does not require VPS36.*
Movie of a vacuole fusion event in a vps36Δ cell demonstrating the formation of intralumenal fragment decorated with Hxt3-GFP after heat stress. Hxt3-GFP and FM4-64 fluorescence, and the two channels merged are shown. Scale bar and time are shown in Figure 3E.

File Name: Supplementary Movie 5.
Description: *Hxt3-GFP internalization by cycloheximide does not require VPS36.*
Movie of a vacuole fusion event in a vps36Δ cell demonstrating the formation of intralumenal fragment decorated with Hxt3-GFP after cycloheximide treatment. Hxt3-GFP and FM4-64 fluorescence, and the two channels merged are shown. Scale bar and time are shown in Figure 4E.
